# Comprehensive Evaluation of Oral Diseases in Patients with Gastrointestinal Cancers: Epidemiological Evidence from a 10-Year Retrospective Study

**DOI:** 10.3390/cancers18121941

**Published:** 2026-06-14

**Authors:** Chiharu Kawamoto, Hirofumi Kaneko, Ryotaro Yago, Yudai Matsuo, Yuto Nakamura, Takuma Mirokuin, Shuhei Hoshika, Hidehiko Sano, Atsushi Tomokiyo, Naoya Sakamoto

**Affiliations:** 1 Department of Operative Dentistry, Hokkaido University Hokkaido University Hospital, Kita 14 Nishi 5, Kita-ku, Sapporo 060-8648, Japan; 2 Department of Restorative Dentistry, Hokkaido University Graduate School of Dental Medicine, Kita 13 Nishi 7, Kita-ku, Sapporo 060-8586, Japan; 3 Department of Restorative Dentistry, Hokkaido University Faculty of Dental Medicine, Kita 13 Nishi 7, Kita-ku, Sapporo 060-8586, Japan; 4 Department of Gastroenterology and Hepatology, Hokkaido University Faculty of Medicine, Kita 15 Nishi 7, Kita-ku, Sapporo 060-8638, Japan

**Keywords:** gastrointestinal cancers, dental caries, apical periodontitis, periodontal disease, DMFT index

## Abstract

**Background:** The association between oral health and gastrointestinal (GI) cancers has been primarily investigated within a periodontitis-centered framework. However, the potential contribution of cumulative oral disease burden, including dental caries and apical pathology, remains insufficiently explored. The Decayed, Missing, and Filled Teeth (DMFT) index reflects lifetime exposure to oral microbial dysbiosis and chronic inflammation. **Methods:** This retrospective exploratory study included patients with GI cancers referred for perioperative oral screening and management at a tertiary care center between 2015 and 2025. Oral health was evaluated using the DMFT index, periodontal probing depth, and radiographically diagnosed apical periodontitis. Age-stratified DMFT and periodontal parameters were compared with national reference data, while apical periodontitis prevalence was descriptively assessed. **Results:** Patients with GI cancers demonstrated higher DMFT values than national averages across most adult age groups. The prevalence of periodontal pockets (≥4 mm and ≥6 mm) was also elevated. Apical periodontitis was common, affecting 46.3% of patients, with some age groups exceeding 50%. Overall, these findings indicate oral disease clustering with coexisting chronic oral conditions. **Conclusions:** Patients with GI cancers exhibit substantial oral disease burden, including increased caries experience, periodontal pathology, and apical lesions. These findings suggest that the oral–gastrointestinal cancer relationship may extend beyond a periodontitis-centered paradigm, and that cumulative oral disease burden—including cariogenic processes—may represent an underrecognized component of this axis. The DMFT index may serve as a surrogate marker of lifelong oral inflammatory exposure. While causal relationships cannot be established, this study provides a basis for future mechanistic and longitudinal investigations.

## Simple Summary

Patients with gastrointestinal (GI) cancer often experience oral problems, but most previous research has focused mainly on gum disease. Dental caries and apical periodontitis are very common oral diseases, yet their relationship with GI cancer has been poorly studied. In this study, we examined the oral health of patients with GI cancer over a ten-year period and compared their dental conditions with national survey data. We found that these patients had more tooth decay, more severe periodontal disease, and a higher prevalence of apical periodontitis than the general population, particularly among middle-aged and older adults. These findings suggest that multiple oral diseases tend to occur together in patients with GI cancer. A better understanding of this relationship may help improve oral care strategies for cancer patients and support future research on how oral health may influence cancer development and outcomes.

## 1. Introduction

Gastrointestinal (GI) cancers are among the leading causes of cancer-related morbidity and mortality worldwide and are increasingly recognized as diseases influenced not only by genetic alterations but also by chronic inflammatory and microbial factors [1,2]. In recent years, growing attention has been directed toward the potential role of the oral cavity in GI carcinogenesis, leading to the emergence of the concept of an “oral–GI axis” [3,4]. Oral microorganisms and chronic oral inflammatory conditions have been proposed to contribute to systemic inflammation, immune modulation, and microbial translocation to the gastrointestinal tract [3,4].

Among oral diseases, periodontal disease has been most extensively investigated in relation to GI malignancies, particularly colorectal and gastric cancers [5,6,7,8]. Several studies have reported associations between periodontitis and increased risk of GI cancers, while periodontal pathogens such as *Fusobacterium nucleatum* have been identified within colorectal tumor tissues [9,10,11]. Emerging evidence further suggests associations between periodontal disease and other GI malignancies, including gastric cancer, potentially mediated through chronic inflammation and systemic dissemination of oral microorganisms [12]. Collectively, these findings have established a largely periodontitis-centered paradigm in oral oncology research.

However, the predominant focus on periodontitis may have contributed to the relative under-recognition of other chronic oral inflammatory conditions. Dental caries is the most prevalent chronic infectious disease worldwide and is characterized by persistent biofilm activity, acid production, and repeated cycles of demineralization and repair [13]. Caries-associated microorganisms, including *Streptococcus mutans* and *Lactobacillus* species, exhibit acidogenicity and varying degrees of acid tolerance [14,15]. Apical periodontitis similarly represents a chronic periapical inflammatory focus capable of releasing microorganisms and inflammatory mediators into the systemic circulation [16,17]. Importantly, large population-based studies have also reported associations between poor dental health and increased risk of gastric cancer in nationwide cohorts [12]. Despite these findings, epidemiological evidence specifically linking dental caries and apical periodontitis with GI cancers remains limited [12,18].

Caries experience is commonly quantified using the DMFT index (Decayed, Missing, and Filled Teeth) [19]. Although widely employed in dental epidemiology, the broader medical significance of DMFT may not be widely recognized in oncology research. Unlike measures reflecting transient infection, DMFT represents a cumulative, largely irreversible record of lifetime exposure to cariogenic biofilms and chronic oral inflammatory processes [20]. The “M” component reflects tooth loss frequently resulting from advanced infection and inflammatory destruction, while the “D” and “F” components indicate active or previously treated caries lesions. Therefore, an elevated DMFT score may serve as a surrogate marker of cumulative oral microbial dysbiosis and long-term inflammatory burden rather than merely current dental decay.

In Japan, the nationwide Survey of Dental Diseases, conducted every five years by the Ministry of Health, Labour and Welfare, provides age-stratified epidemiological benchmarks for caries experience and periodontal status across the general population [21]. Such structured and periodically updated national oral health datasets are not uniformly available in many countries. The availability of these standardized reference data offers a unique opportunity to contextualize oral health findings in specific patient populations against representative national standards.

Based on our long-term clinical observations in perioperative oral management, we frequently encountered untreated caries, apical lesions, and advanced periodontal pockets in patients with GI cancers. These findings appeared to exceed what would be expected for age alone and raised the possibility that cumulative oral inflammatory burden might be characteristic of this population.

Despite increasing interest in the oral–GI axis, most epidemiological studies have focused primarily on periodontal disease, particularly its association with colorectal cancer [5,6,7,8]. Accordingly, other chronic oral inflammatory conditions—such as dental caries and apical periodontitis—have received relatively limited attention in the context of GI malignancies, and comprehensive evaluations incorporating multiple oral disease indicators remain scarce, particularly when benchmarked against population-based national oral health datasets. Addressing this gap may improve understanding of cumulative oral inflammatory burden in patients with GI cancers and help clarify whether oral disease clustering extends beyond periodontitis alone.

We hypothesized that patients with GI cancers exhibit not only periodontal pathology but also elevated cumulative caries burden and apical pathology compared with age-matched national standards. Therefore, the present retrospective study comprehensively evaluated the oral condition of patients with GI cancers using three indicators: (1) caries experience assessed by the DMFT index, (2) periodontal pocket depth, and (3) radiographically diagnosed apical periodontitis. Caries and periodontal findings were compared with nationwide survey data, while apical periodontitis prevalence was evaluated in the context of existing reports. Through this approach, we aimed to re-examine the oral–GI cancer association from a broader perspective extending beyond the traditional periodontitis-centered framework and to inform future mechanistic and longitudinal studies.

## 2. Materials and Methods

### 2.1. Study Population

**Figure 1:**
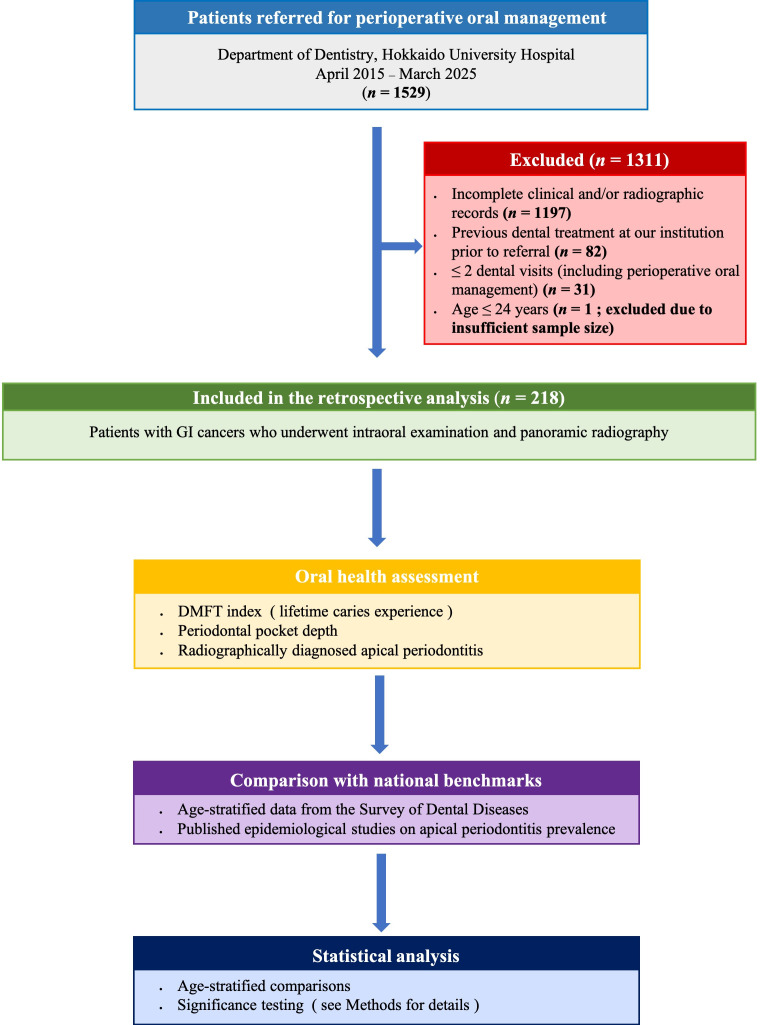
Study flow diagram of patient selection and analysis. This diagram illustrates the patient selection process for the retrospective analysis. Patients referred for perioperative oral management during the study period were screened, and predefined exclusion criteria were applied. A total of 218 patients with GI cancers were included in the final analysis. Oral health parameters were assessed and compared with age-stratified national survey data and published epidemiological studies.

This retrospective exploratory study included patients with GI cancers who were routinely referred to the Department of Dentistry, Hokkaido University Hospital, for perioperative oral screening and management as part of institutional perioperative care protocols between April 2015 and March 2025. At our institution, patients with GI cancers are routinely referred for perioperative oral assessment regardless of oral symptoms or dental complaints. Therefore, the study cohort represents consecutive GI cancer patients undergoing perioperative evaluation rather than a subgroup selected because of poor oral health. A total of 1529 patients were initially identified, of whom 1311 were excluded based on predefined criteria, resulting in a final analytical sample of 218 patients. Among consecutive cases, patients who underwent both intraoral examination and panoramic radiography at their initial visit were included. The inclusion and exclusion process is summarized in Figure 1. Patients were excluded if they had received dental treatment at our institution prior to referral, in order to reduce potential referral bias associated with pre-existing dental complaints or ongoing dental treatment, had incomplete clinical or radiographic records, or had an insufficient number of perioperative visits for adequate assessment. Patients aged 15–24 years were also excluded from statistical analyses due to the extremely small sample size. Cancer sites were classified according to predefined categories (Table 1), based on the primary lesion when multiple tumors were present. Because this was an exploratory retrospective study, no a priori sample size calculation was performed. The sample size was determined by all eligible consecutive patients during the study period, and the findings should be interpreted as hypothesis-generating.

### 2.2. Age Groups, Patient Profiles and Reference Data

Patients were stratified into the following age groups according to the 2022 National Survey of Dental Diseases: 25–34, 35–44, 45–54, 55–64, 65–74, and ≥75 years. Additionally, patient profiles examined gender, cancer stage, presence or absence of chemotherapy, presence or absence of radiation therapy, smoking history, history of diabetes, and whether the cancer was initial or recurrent.

(1) DMFT index and periodontal pockets: Compared with age-stratified data from the national survey.
(2) Apical periodontitis: Since no corresponding data are available in the national survey, prevalence was compared with previously published domestic and international epidemiological studies.

### 2.3. Clinical Assessments

All statistical analyses were performed using SPSS Statistics version 28.0 (IBM, Armonk, NY, USA). The 15–24 age group (*n* = 1) was omitted from statistical analyses because the small sample size did not allow for reliable statistical evaluation.

**(1)**  **Caries** **status (DMFT index)**

Based on dental records and panoramic radiographs at the initial visit, decayed (D), missing (M), and filled (F) teeth were recorded, and the DMFT index was calculated. Root caries and secondary caries were also included as decayed teeth. DMFT values were analyzed as continuous variables. The DMFT index was interpreted as an epidemiological measure of cumulative lifetime caries experience and oral disease burden rather than a marker of current inflammatory activity. For each age group, Welch’s *t*-test was used to compare mean DMFT values between cancer patients and the national survey. Statistical significance was set at *p* < 0.05.

**(2)**  **Apical Periodontitis**

Apical periodontitis was defined, in accordance with previous epidemiological studies using the Periapical Index (PAI), as a lesion with a PAI score ≥ 3, corresponding to a clearly identifiable periapical radiolucency [22,23]. Two dentists independently evaluated the images, and discrepancies were resolved by discussion. Prevalence was summarized by age group. Because no national survey data exist for apical periodontitis and an appropriate non-cancer control cohort with comparable radiographic records was not available, statistical comparisons were not performed; instead, prevalence was evaluated descriptively and interpreted as exploratory information. The Wilson score method was used to estimate prevalence, with 95% confidence intervals calculated. The mean number of periapical lesions per patient was calculated by age, and the Poisson distribution was used to estimate 95% confidence intervals.

**(3)**  **Periodontal Status**

Sites with periodontal probing depths of ≥4 mm and ≥6 mm were recorded as binary variables. These prevalences were compared with age-stratified national survey data using chi-square tests. Fisher’s exact test was applied when expected frequencies were <5. Statistical significance was set at *p* < 0.05. In addition, maximum probing depth, mean probing depth across all teeth, and the proportion of sites ≥4 mm for each patient were summarized descriptively. Because equivalent indices are not reported in the national survey, no formal statistical comparisons were performed for these parameters.

### 2.4. Statistical Analysis

This study was approved by the Ethical Review Board for Life Science and Medical Research, Hokkaido University Hospital (approval number: 021-0216). As a retrospective observational study not involving human biological samples, the requirement for individual informed consent was waived in accordance with the Ethical Guidelines for Medical and Biological Research Involving Human Subjects in Japan. Instead, study information was disclosed on the hospital website, and patients or their representatives were given the opportunity to opt out. All analyses were conducted using anonymized data.

## 3. Results

### 3.1. Patient Profiles

Patients included in this study ranged in age from 25 to over 90 years and were classified into seven age groups according to the National Survey of Dental Diseases. Details of the patient profiles are shown in Table 2.

### 3.2. DMFT Index

**Figure 2:**
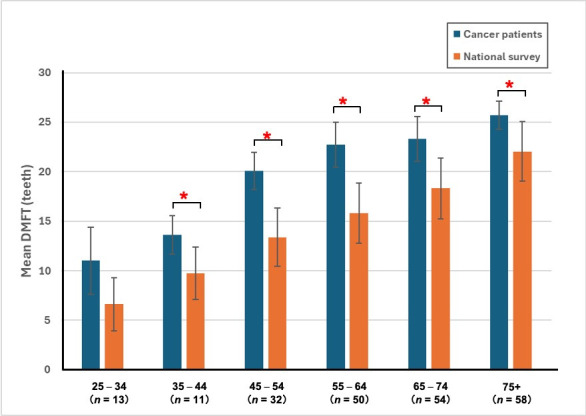
Comparison of mean DMFT between GI cancer patients and national survey data. Mean numbers of decayed, missing, and filled teeth (DMFT) are presented according to age group. Blue bars indicate patients with GI cancers (*n* shown below each group, error bars represent standard deviations), and orange bars indicate the 2022 national dental survey. Asterisks (*) above brackets denote statistically significant differences between GI cancer patients and the national survey within the corresponding age group (Welch’s *t*-test, *p* < 0.05).

The mean DMFT values of patients with GI cancers in each age group and the corresponding national averages are shown in Figure 2.

Across all age groups, the mean DMFT of patients with GI cancers were consistently higher than the national averages. Statistically significant differences were observed in all groups aged ≥ 25 years (*p* < 0.05, Welch’s *t*-test). In the 25–34 age group, the mean DMFT was 11.0 ± 6.8, significantly higher than the national average of 6.3 (*p* = 0.014). The 35–44 age group showed 13.6 ± 3.9 vs. 9.6 (*p* = 0.003). The 45–54 age group demonstrated 20.1 ± 3.7 vs. 13.4 (*p* < 0.001), the 55–64 age group 22.7 ± 4.6 vs. 15.7 (*p* < 0.001), the 65–74 age group 23.3 ± 4.5 vs. 18.2 (*p* < 0.001), and the ≥75 age group 25.7 ± 2.9 vs. 21.8 (*p* < 0.001). Taken together, these findings demonstrate a marked accumulation of caries experience among patients with GI cancers aged 25 years and older compared with the general population.

### 3.3. Apical Periodontitis

**Figure 3:**
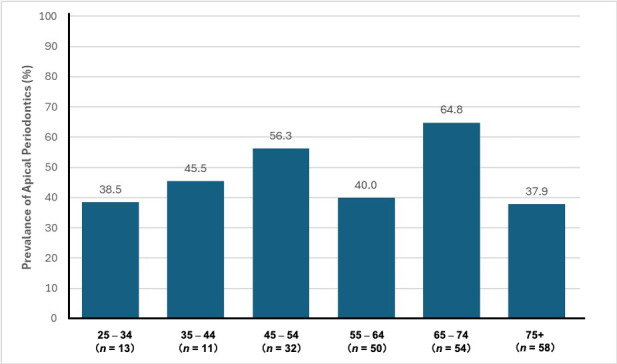
Prevalence of apical periodontitis in GI cancer patients. The prevalence of apical periodontitis (≥3 PAI) is shown according to age group. Bars represent the proportion of patients diagnosed with at least one apical lesion in each group. Error bars represent Wilson 95% confidence intervals.

The prevalence of apical periodontitis is shown in Figure 3.

Overall, 46.3% of patients were diagnosed with at least one apical lesion. Age-specific prevalence rates were 38.5% in the 25–34 age group, 45.5% in the 35–44 age group, 56.3% in the 45–54 age group, 40.0% in the 55–64 age group, 64.8% in the 65–74 age group, and 37.9% in the ≥75 age group. Notably, more than half of the patients in the 45–54 and 65–74 age groups presented with apical periodontitis.

**Figure 4:**
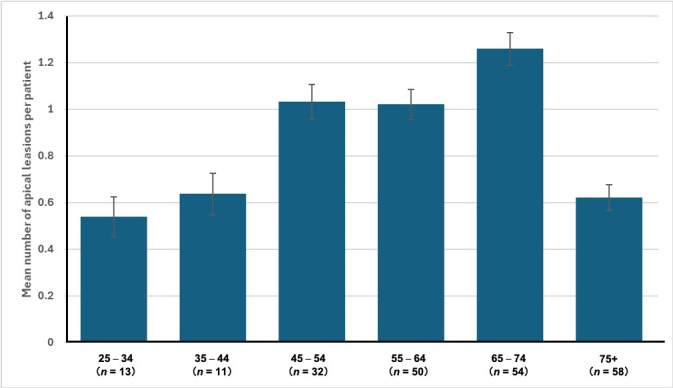
Mean number of apical lesions per cancer patient. The mean number of apical lesions per patient is shown according to age group. Bars indicate patients with GI cancers, and error bars represent 95% confidence intervals based on a Poisson distribution.

The mean number of apical lesions per patient is presented in Figure 4.

The mean number of apical lesions per patient was 0.54 in the 25–34 age group, 0.64 in the 35–44 age group, 1.03 in the 45–54 age group, 1.02 in the 55–64 age group, 1.26 in the 65–74 age group, and 0.62 in the ≥75 age group. In all age groups ≥ 25 years, some patients presented with multiple lesions, and the mean number exceeded one lesion per patient in the 45–54 and 65–74 age groups.

### 3.4. Periodontal Pockets

#### 3.4.1. Prevalence of Periodontal Pockets (≥4 mm and ≥6 mm)

**Figure 5:**
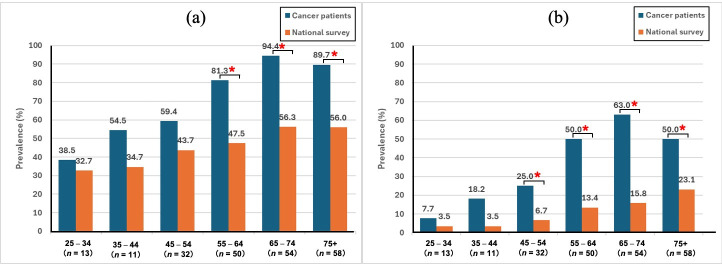
Prevalence of periodontal pockets in patients with GI cancers compared with the national survey. (**a**) Prevalence of periodontal pockets ≥4 mm according to age group. (**b**) Prevalence of periodontal pockets ≥6 mm according to age group. Blue bars indicate patients with GI cancers (*n* values shown below each group), and orange bars indicate the 2022 National Survey of Dental Diseases. Asterisks (*) indicate statistically significant differences between groups. Statistical comparisons were performed using the chi-square test or Fisher’s exact test, as appropriate. Fisher’s exact test was applied when expected frequencies were <5. A *p*-value < 0.05 was considered statistically significant.

The prevalence of periodontal pockets ≥4 mm and ≥6 mm by age group is presented in Figure 5.

In Figure 5a, the prevalence of periodontal pockets ≥4 mm was consistently higher in patients with GI cancer than in the national survey across all age groups. Statistically significant differences were observed in the 55–64 age (78.0% versus 47.5%), 65–74 age (94.4% versus 56.2%), and ≥75 (89.7% versus 56.0%) age groups (all *p* < 0.001). Although prevalence was higher in the 25–54 age groups, these differences did not reach statistical significance. Fisher’s exact test was applied for comparisons involving small expected frequencies.

In Figure 5b, the prevalence of periodontal pockets ≥6 mm, indicative of severe periodontitis, was markedly higher in GI cancer patients than in the general population. Significant differences were found in the 45–54 (25.0% versus 6.7%), 55–64 (48.0% versus 13.4%), 65–74 (63.0% versus 15.8%), and ≥75 (50.0% versus 23.1%) age groups (all *p* < 0.001). Differences in the 25–44 age groups were not statistically significant. Fisher’s exact test was applied when expected frequencies were small.

#### 3.4.2. Further Analysis of Probing Depth

**Figure 6:**
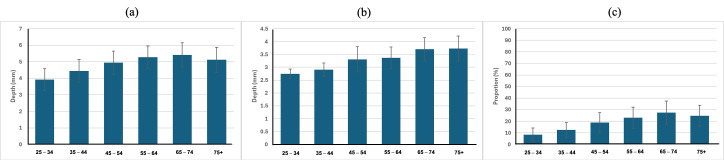
Further analysis of periodontal pocket depth in patients with GI cancers. (**a**) Maximum probing depth per patient; (**b**) mean probing depth across all teeth per patient; and (**c**) proportion of sites with probing depth ≥4 mm per patient. Bars denote patients with GI cancers; error bars indicate standard deviations (SDs).

A further analysis of probing depth distribution is shown in Figure 6.

High values were observed for maximum probing depth, mean probing depth, and the proportion of sites ≥4 mm, indicating a tendency toward severe periodontitis throughout the patient group. These parameters were markedly elevated in middle-aged and older patients, with mean maximum probing depths exceeding 6 mm and the proportion of sites ≥4 mm surpassing 35% in the 65–74 age group.

## 4. Discussion

**Figure 7:**
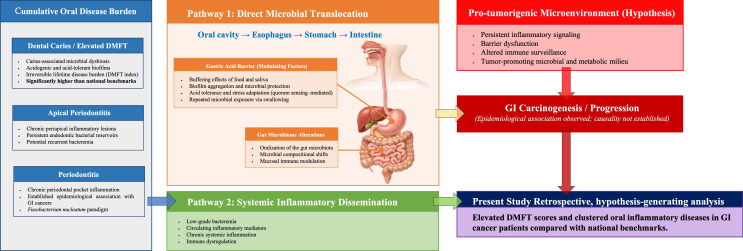
Proposed caries-centered oral–GI cancer axis. This schematic illustrates a conceptual framework extending beyond the traditional periodontitis-centered paradigm. Cumulative oral disease burden—reflected by elevated DMFT (caries experience), apical periodontitis, and marginal periodontitis—may influence the GI environment through two non-mutually exclusive pathways: (1) direct microbial translocation facilitated by buffering conditions, biofilm protection, and quorum sensing–mediated stress adaptation; and (2) systemic inflammatory dissemination via recurrent bacteremia and circulating inflammatory mediators. The present study provides hypothesis-generating epidemiological evidence of increased cumulative caries burden and clustering of oral diseases in patients with GI cancers compared with national benchmarks. Causal relationships remain to be established.

Based on the present findings, we propose a conceptual model describing a potential caries-centered oral–GI cancer axis (Figure 7).

### 4.1. Principal Findings

Compared with the national reference data, patients with GI cancers in the present cohort demonstrated a greater burden of oral diseases, including dental caries experience, periodontal pathology, and apical periodontitis. However, because the study design was retrospective and comparisons were made with aggregate population data, these findings should be interpreted as descriptive and hypothesis-generating rather than evidence of an independent association. The present study provides a novel perspective by shifting the conventional periodontitis-centered paradigm toward a broader framework that incorporates cumulative caries burden and apical pathology. While previous studies have primarily focused on periodontal disease, our findings suggest that oral disease clustering—including caries experience quantified by the DMFT index—may represent an underrecognized component of the oral–GI cancer axis. This integrative approach offers a new conceptual model for understanding the cumulative impact of oral inflammatory conditions on systemic disease. Across nearly all adult age groups, the DMFT index significantly exceeded national averages, indicating substantial lifetime caries experience. Similarly, periodontal pockets ≥4 mm and ≥6 mm were more prevalent than in the general population, particularly among middle-aged and older individuals. Furthermore, apical periodontitis was highly prevalent, with more than half of patients in certain age groups affected. Because an appropriate non-cancer control cohort with comparable radiographic records was not available, apical periodontitis was evaluated descriptively and should be interpreted as exploratory information rather than evidence of a statistically confirmed association. Consistent with previous evidence, including systematic reviews and meta-analyses demonstrating associations between periodontal disease, oral pathogens, and colorectal cancer risk [6,7,8,24,25,26,27,28], our findings extend this concept by suggesting that oral disease clustering—including caries experience and apical pathology—may also contribute to the oral–GI cancer axis.

### 4.2. Reframing the Oral–GI Cancer Axis

Oral microbial dysbiosis has been increasingly recognized as a systemic modifier of carcinogenesis, with several oral pathogens capable of influencing tumor development through inflammatory, immune, and metabolic pathways [9,24,26]. Among these, *Fusobacterium nucleatum* has been frequently implicated in colorectal carcinogenesis and may translocate from the oral cavity to the intestinal environment, potentially via hematogenous routes [9,10,11]. To date, the oral–GI cancer axis has been largely discussed within a periodontitis-centered framework, with numerous studies focusing on periodontal pathogens and their association with colorectal cancer [24]. While this paradigm has provided valuable insights, it has also generated important mechanistic understanding of microbe–host interactions; however, an exclusive emphasis on periodontitis may overlook other chronic inflammatory sources within the oral cavity. In particular, such a framework may not fully capture the complexity of oral microbial ecology and its systemic implications. In this context, the present findings support a broader conceptual framework in which multiple chronic oral conditions—including dental caries and apical periodontitis—collectively contribute to long-term microbial and inflammatory exposure, suggesting that the oral–GI cancer relationship may be better understood as a cumulative and multifactorial process rather than one driven by a single disease entity. Dental caries and apical periodontitis represent persistent microbial challenges characterized by long-term biofilm activity, recurrent tissue destruction, and chronic inflammatory stimulation [29]. Unlike periodontal parameters such as probing depth, which primarily reflect current disease status, caries experience quantified by the DMFT index reflects cumulative lifetime exposure to cariogenic dysbiosis. The consistently elevated DMFT values observed in this cohort raise the possibility that cumulative caries burden constitutes an underrecognized component of the oral–GI cancer relationship.

Importantly, DMFT is not merely a count of decayed teeth. The “M” component reflects tooth loss frequently resulting from advanced infection and chronic inflammatory destruction, while the “D” and “F” components represent ongoing or past cariogenic biofilm activity. Thus, a high DMFT score may serve as an epidemiological indicator of cumulative lifetime oral disease experience and its long-term consequences rather than transient dental decay alone.

### 4.3. DMFT as a Marker of Cumulative Oral Exposure

A notable strength of this study lies in the availability of nationally standardized oral health data in Japan. The Survey of Dental Diseases, conducted every five years by the Ministry of Health, Labour and Welfare, provides age-stratified epidemiological benchmarks across the general population [21]. Such structured, regularly updated nationwide datasets are not uniformly available in many countries.

By comparing our cohort with these national standards, the observed elevation in DMFT and periodontal parameters can be contextualized against a representative population baseline. This comparison reduces reliance on small control groups and enhances interpretability, although residual confounding cannot be excluded. Notably, a central finding of this study is the consistent elevation of the DMFT index across age groups. In contrast to periodontal parameters, which primarily reflect current inflammatory status and are sensitive to short-term changes, the DMFT index represents a cumulative and largely irreversible record of lifelong exposure to cariogenic biofilms and chronic oral inflammation [30]. The “M” component reflects tooth loss often resulting from advanced infection, while the “D” and “F” components indicate active and past disease experience.

From this perspective, DMFT may function not merely as a dental index but as an integrative epidemiological indicator of cumulative lifetime oral disease experience. The observed elevation in DMFT among patients with GI cancers suggests that long-term exposure to such conditions—rather than transient oral disease—may be associated with an increased risk of GI cancers.

Importantly, DMFT integrates the effects of diverse determinants, including oral hygiene behavior, dietary patterns, smoking, and systemic conditions such as diabetes, as well as environmental influences. While these factors may act as confounders, they also reinforce the interpretation of DMFT as an integrative indicator of lifetime exposure, capturing the combined impact of multiple risk factors over time.

### 4.4. Contextual Interpretation Using National Reference Data

A notable strength of this study is the use of age-stratified national reference data from Japan. The nationwide Survey of Dental Diseases provides standardized epidemiological benchmarks that enable contextualization of oral health findings against a representative population [21]. In Japan, school-based dental examinations are conducted throughout childhood and adolescence, and universal health coverage reduces financial barriers to dental care [31]. These structural features support relatively broad access to dental services across socioeconomic groups. Despite these advantages, the study cohort demonstrated consistently elevated DMFT and periodontal parameters compared with national averages. This finding suggests that the observed differences are unlikely to be fully explained by disparities in access to care alone and may instead reflect underlying biological, behavioral, or systemic factors. Nevertheless, direct comparability remains limited, and residual confounding—particularly related to socioeconomic status and lifestyle—cannot be excluded.

### 4.5. Potential Biological Mechanisms

The association between elevated caries burden and GI cancers may be conceptualized through two non-mutually exclusive pathways.

#### 4.5.1. Direct Microbial Translocation

Recent advances in molecular microbiology further support the adaptive capacity of cariogenic bacteria [32]. Quorum sensing (QS) systems in Streptococcus mutans, including the comCDE regulatory pathway, coordinate population-level behaviors such as biofilm formation, stress responses, and regulation of acid tolerance [33]. Through QS-mediated gene regulation, bacterial communities may enhance bacterial resilience under hostile and fluctuating conditions [34].

Streptococcus mutans, a well-known cariogenic bacterium, exhibits both acidogenicity and acid tolerance, being metabolically active at around pH 4 [35,36], although survival is difficult at pH 3.5 for extended periods [37]. Although many oral bacteria are highly sensitive to gastric acidity, brief survival in the stomach cannot be discounted, since gastric pH varies with food intake and the buffering action of saliva. Food boluses, mucus layers, and biofilm structures may also offer temporary protection from acid exposure [15]. Consistent with these possibilities, S. mutans has been detected in the intestine [14].

Although direct evidence that QS enables sustained survival through gastric acidity is limited, this coordinated stress adaptation provides a biological rationale for considering transient survival and passage of cariogenic biofilm fragments during periods of higher gastric pH and buffering by food and saliva [14,15,38].

#### 4.5.2. Systemic Inflammatory Dissemination

Chronic caries and apical lesions can release bacteria and inflammatory mediators into the systemic circulation [16,17,39]. Recurrent low-grade bacteremia and sustained cytokine exposure may contribute to systemic inflammatory priming [40]. Given that chronic inflammation is a recognized driver of carcinogenesis, cumulative oral inflammatory burden may modulate tumor-promoting environments through immune dysregulation or altered host–microbe interactions [41].

These pathways remain hypothetical; however, they provide a biologically plausible framework linking cumulative caries burden with GI malignancies.

### 4.6. Clinical Implications

From a clinical perspective, the findings suggest that patients with GI cancers frequently present with substantial untreated or cumulative oral disease. Moreover, these findings indicate that such oral disease burden is not limited to a single condition but is multifactorial in nature, encompassing dental caries, apical pathology, and periodontal disease. If future longitudinal studies validate these observations, DMFT assessment—an inexpensive and readily obtainable parameter—may serve as a clinically accessible and practical surrogate marker to identify individuals with elevated cumulative oral inflammatory exposure. In this context, the DMFT index may provide a practical tool for identifying individuals with elevated cumulative oral inflammatory burden in routine clinical settings.

Consistent with previous clinical reports demonstrating a high prevalence of severe periodontitis among patients with gastric cancer [8,12,28], our findings further suggest that oral disease burden in this population extends beyond periodontal pathology to include cumulative caries experience and apical disease. These findings reinforce the importance of comprehensive oral evaluation, in which caries and apical pathology are considered alongside periodontal status as part of an integrated assessment of oral health.

Taken together, these findings indicate that cumulative oral disease burden—including caries experience and apical pathology—was frequently observed among patients with GI cancers. Although causality cannot be inferred, these observations warrant further investigation in longitudinal, mechanistic, and multicenter studies.

### 4.7. Limitations

This study has several limitations.

First, this was a single-center retrospective study of hospital-based patients with GI cancers, which may limit generalizability. Second, comparisons were performed against aggregate national survey data rather than individual-level controls. Therefore, adjustment for important confounders, including smoking status, alcohol consumption, dietary habits, socioeconomic status, and access to dental care, was not possible. Third, the cross-sectional nature of the analysis precludes assessment of temporality and causality. Reverse causation cannot be excluded because oral health status may have been influenced by behavioral, nutritional, or systemic changes occurring before cancer diagnosis.

Additionally, the small number of younger patients restricts interpretation in this age group. Finally, the absence of microbiological analyses limits mechanistic insights. Therefore, these findings should be interpreted as hypothesis-generating rather than confirmatory. Furthermore, DMFT is an epidemiological measure of cumulative oral disease experience and should not be interpreted as a direct microbiological or inflammatory biomarker.

### 4.8. Future Directions

Future research should incorporate multicenter prospective cohorts and detailed adjustment for socioeconomic and lifestyle factors. Integration of fecal microbiome sequencing, metagenomic analyses, and longitudinal tracking of oral–gut microbial dynamics will be essential to clarify whether cumulative caries burden contributes directly to GI carcinogenesis or represents a surrogate marker of systemic vulnerability. Elucidating these mechanisms may open new avenues for interdisciplinary prevention strategies bridging dentistry and oncology.

To further contextualize the implications of this study, a brief SWOT analysis is provided below. From a SWOT perspective, the strengths of this study include the use of a standardized national reference dataset and comprehensive evaluation of multiple oral disease indicators. Weaknesses include its retrospective design, potential selection bias, and lack of socioeconomic and microbiological data. Opportunities lie in future prospective multicenter studies integrating microbiome analyses. Threats include the complexity of establishing causal relationships in the oral–GI axis and the potential influence of unmeasured systemic and environmental factors that may confound observed associations.

## 5. Conclusions

In conclusion, this study demonstrates that patients with GI cancers are characterized by oral disease clustering, including elevated caries experience, periodontal pathology, and apical lesions. These findings support a broader conceptual framework of the oral–GI cancer axis that extends beyond a periodontitis-centered paradigm and further suggest that cumulative oral disease burden—including cariogenic processes—may represent an underrecognized component of this relationship. While causal inference is not possible, this perspective highlights the importance of considering oral health as a lifelong and multifactorial systemic exposure. These findings may inform future research and support a more comprehensive approach to oral health assessment in patients with GI cancers.

## Figures and Tables

**Table 1:** Major Categories of GI Cancer for Analysis.

Major Category	Representative Diseases
Pharyngeal cancer	Oropharyngeal cancer, Hypopharyngeal cancer, Nasopharyngeal cancer
Esophageal cancer	Esophageal cancer
Gastric cancer	Cardia cancer, Gastric body cancer, Pyloric cancer
Duodenal cancer	Duodenal papillary cancer, Proximal duodenal cancer, Distal duodenal cancer
Colorectal cancer	Colon cancer (Ascending, Transverse, Descending, Sigmoid), Rectal cancer
Pancreatic cancer	Pancreatic head cancer, Pancreatic body/tail cancer
Liver cancer	Hepatocellular carcinoma, Intrahepatic cholangiocarcinoma (also called cholangiocellular carcinoma)
Biliary tract cancer	Gallbladder cancer, Extrahepatic cholangiocarcinoma (hilar cholangiocarcinoma, distal cholangiocarcinoma)

**Table 2:** Patient profiles.

Category	Details (*n*)
Age	25–34 (13), 35–44 (11), 45–54 (32), 55–64 (50), 65–74 (54), 75+ (58)
Sex	Male (126), Female (92)
Age and Sex (Male/Female)	25–34 (3/10), 35–44 (6/5), 45–54 (10/22), 55–64 (32/28), 65–74 (40/14), 75+ (35/23)
Cancer Site	Pharyngeal cancer (33), Esophageal cancer (23), Gastric cancer (31), Duodenal cancer (5), Colorectal cancer (67), Pancreatic cancer (27), Liver cancer (17), Biliary tract cancer (15)
Cancer Stage	Stage I (40), Stage II (46), Stage III (46), Stage IV (86)
Chemotherapy	Yes (96), No (122)
Radiation therapy	Yes (29), No (189)
Smoking history	Yes (122), No (96)
Diabetes mellitus	Yes (44), No (174)
Initial/Recurrent	Initial (180), Recurrent (38)

## Data Availability

The original contributions presented in this study are included in the article. Further inquiries can be directed to the corresponding author.
